# Effect of the initial energy layer and spot placement parameters on IMPT delivery efficiency and plan quality

**DOI:** 10.1002/acm2.13997

**Published:** 2023-04-26

**Authors:** Mingyao Zhu, Stella Flampouri, Alex Stanforth, Roelf Slopsema, Zachary Diamond, William LePain, Katja Langen

**Affiliations:** ^1^ Department of Radiation Oncology Emory University School of Medicine Atlanta Georgia USA; ^2^ Mechanical Engineering, Nuclear Radiological Engineering & Medical Physics Georgia Institute of Technology Atlanta Georgia USA; ^3^ Emory Healthcare Atlanta Georgia USA

**Keywords:** dose optimization, proton therapy, treatment planning

## Abstract

**Purpose:**

Improving efficiency of intensity modulated proton therapy (IMPT) treatment can be achieved by shortening the beam delivery time. The purpose of this study is to reduce the delivery time of IMPT, while maintaining the plan quality, by finding the optimal initial proton spot placement parameters.

**Methods:**

Seven patients previously treated in the thorax and abdomen with gated IMPT and voluntary breath‐hold were included. In the clinical plans, the energy layer spacing (ELS) and spot spacing (SS) were set to 0.6–0.8 (as a scale factor of the default values). For each clinical plan, we created four plans with ELS increased to 1.0, 1.2, 1.4, and SS to 1.0 while keeping all other parameters unchanged. All 35 plans (130 fields) were delivered on a clinical proton machine and the beam delivery time was recorded for each field.

**Results:**

Increasing ELS and SS did not cause target coverage reduction. Increasing ELS had no effect on critical organ‐at‐risk (OAR) doses or the integral dose, while increasing SS resulted in slightly higher integral and selected OAR doses. Beam‐on times were 48.4 ± 9.2 (range: 34.1–66.7) seconds for the clinical plans. Time reductions were 9.2 ± 3.3 s (18.7 ± 5.8%), 11.6 ± 3.5 s (23.1 ± 5.9%), and 14.7 ± 3.9 s (28.9 ± 6.1%) when ELS was changed to 1.0, 1.2, and 1.4, respectively, corresponding to 0.76–0.80 s/layer. SS change had a minimal effect (1.1 ± 1.6 s, or 1.9 ± 2.9%) on the beam‐on time.

**Conclusion:**

Increasing the energy layers spacing can reduce the beam delivery time effectively without compromising IMPT plan quality; increasing the SS had no meaningful impact on beam delivery time and resulted in plan‐quality degradation in some cases.

## 1 INTRODUCTION

Proton beam therapy (PBT) provides better normal tissue sparing than traditional photon radiation therapy, owing to the zero exit‐dose beyond the Bragg peaks. The pencil beam scanning (PBS) technique further improves the normal tissue sparing through intensity modulation.^1^ PBS has therefore gained popularity and become delivery method of choice in most proton treatment facilities.^2^

Despite a continuing increase in the number of the proton treatment centers,^2^ PBT is still a relative sparse resource, compared to the traditional photon radiation therapy. Improving treatment efficiency can allow more patients to receive proton therapy. One approach to improve the treatment efficiency is to reduce the beam delivery time. Shorter treatment duration will not only increase the patient throughput, but also improve patient comfort, especially for patients with large tumors associated with longer treatment times and for treatments that use breath‐hold. For multiple room centers, shorter beam delivery time in any treatment room also reduces wait time for other rooms that are in the beam queue.

Because of the dynamic nature of the IMPT plan delivery, motion interplay must be considered when treating moving anatomies.^3^, ^4^ Breath‐hold is a desired motion management technique to remove motion and the dosimetric degradation caused by the interplay effect. Most patients can only hold their breath for a limited time duration,^5^ typically about 30 s, so each treatment beam may be delivered through multiple breath‐hold cycles. Reducing the beam delivery time can decrease the number of breath‐hold cycles needed, and further improve the treatment efficiency. Furthermore, delivering each beam within one breath‐hold can avoid dose degradation caused by potential anatomy variation between multiple breath‐holds.

For PBS, the beam delivery time is affected by multiple factors.^6^ For a specific target and prescribed dose per fraction, the beam delivery time changes with the beam current into the treatment head (nozzle), energy layer switch time, spot switch time, number of energy layers and spots, and dose (MU) per spot. For a proton treatment machine using a cyclotron accelerator, the nozzle current usually varies with beam energy and is set by the minimum spot weight (MU) within the energy layer. It has been reported that increasing the minimum spot MU can reduce the beam delivery time.^7^, ^8^

Further reduction of beam delivery time can be achieved by reducing the number of energy layers, as the total layer switch time increases with a number of layers. Using more energy layers and spots for dose optimization, in principle, allows more freedom of plan optimization, but sometimes inverse optimized plans can be unnecessarily complex.^9^ For IMPT plans, previous studies have demonstrated that reducing the number of proton energy layers or the number of proton spots can shorten the delivery time while maintaining the same plan quality.^10^, ^11^, ^12^, ^13^

One challenge in optimizing the number of energy layers or proton spots is that the commercial treatment planning systems are not capable of varying the energy layers or proton spots in the plan optimization process. Instead, initial proton spots with predefined energy layer spacing (ELS) and spot spacing (SS) are generated before the optimization; only the weights of the individual spots are optimized based on the desired dosimetric objectives. The low‐weight spots are usually filtered during the optimization process^14^ or post‐processed after the optimization is completed^15^ to ensure plan deliverability. Directly optimizing the number of energy layers or proton spots requires advanced research software developed by individual institutions,^10^, ^11^, ^12^, ^13^ that is, currently not available in the routine clinical treatment planning workflow.

The number of energy layers and spots can be varied by setting the ELS and SS values before the optimization process: larger spacing results in fewer energy layers and spots, and vice versa. The purpose of this work is to investigate the effect of varying the initial energy layer and proton SS values on the plan quality and beam delivery time; and seek to improve the treatment efficiency without plan quality degradation.

## 2 METHOD AND MATERIALS

### 2.1 Patient data

This study included data from seven patients previously treated with PBS proton beam therapy for lung (two patients), esophagus (two patients), and liver (three patients) tumors. All patients were simulated with a Siemens CT scanner and a slice thickness of 1.5 mm. To mitigate the respiratory motion, voluntary breath‐hold (SDX by DYN'R Medical Systems) technique was utilized during CT simulation. Each patient was first trained to hold the breath three times for at least 30 s each to assess the feasibility of breath‐hold, and then three consecutive simulation CT images were acquired to evaluate the potential anatomical variation among different breath‐holds. The clinical target volume (CTV) varied from 118 to 360 cm^3^ and the dose per fraction ranged from 1.8 to 3.87 GyRBE. In this study, the GyRBE is defined as the physical dose Gy multiplied by a constant RBE factor of 1.1. Three to five beams were used in the clinical plans. Details of the patient data are listed in Table 1.

**TABLE 1:** Treatment site, clinical target volume (CTV), dose per fraction, total prescribed dose, number of beams, energy layer spacing (ELS), number of energy layers (EL), and spot spacing (SS) data in the clinical plans for the seven patients. The ELS and SS are a scale factor applied to the automatic calculated value by the TPS

Pt.	Site	CTV (cc)	D/fx (Gy)	D (Gy)	N. beams	ELS	N. EL	SS
1	Esophagus	235	1.8	50.40	3	0.6	52 49 43	0.6
2	Liver	210	3.5	52.50	3	0.7	42 36 38	0.7
3	Esophagus	165	2	50.00	5	0.6	45 34 35 50 44	0.6
4	Liver	160	3.87	58.05	3	0.7	27 27 26	0.7
5	Lung	360	2	60.00	5	0.8	37 34 34 35 36	0.8
6	Liver	118	3.87	58.05	3	0.7	22 25 24	0.7
7	Lung	132	2	70.00	4	0.7	38 40 40 33	0.7

### 2.2 Treatment planning

All seven patients were treated with PBS proton plans created in a commercial treatment planning system RayStation Version 9A (RaySearch Laboratories) and delivered with auto‐gating on a ProBeam (Varian Medical System) proton treatment machine with a nominal 4 mm spot size (sigma in air) and an energy range of 70−242 MeV. All plans were optimized such that the prescribed dose covered at least 98% of the CTV volumes, while keeping the OARs and normal tissue doses as low as possible. Three to five fields were selected depending on the target location and other clinical considerations. Each field was optimized to deliver a uniform dose to the target; when the target was split, each portion of the target was covered by at least two beams.

All plans were robustly optimized with 21 scenarios, accounting for 5 mm isocenter shifts in the six cardinal directions and a proton range uncertainty of ±3.5% (±5% for lung patients). Monte Carlo dose calculation was used in both the optimization and final dose calculation; 10 000–20 000 ions/spot were used in the initial spot dose calculation (for optimization) and statical uncertainties of ≤0.5% were used in the final dose calculation. Even though the machine limit is 1.1 MU per proton spot, all clinical and investigated plans were optimized with a minimum spot weight of 3 MU, to reduce the beam delivery time. For the ProBeam system, 1 MU corresponds to 1 cGy/mm^2^ at depth of 2 cm in water for each mono‐energetic layer.

### 2.3 Initial spot placement parameters

In RayStation treatment planning system, the default nominal spot placement parameters, that is, the ELS and SS, are automatically calculated based on the proton energy of each layer.^14^ Specifically, ELS between the two adjacent layers equals the width of the Bragg peak (at 80% intensity) of the higher energy layer in the pair; the SS is 1.06 times the projected spot sigma of the energy layer. Both the ELS and the SS can be scaled by user‐specified factors, providing the flexibility of using more (or less) layers and spots than the default settings. In the seven clinic plans, our typical clinical value for the ELS and SS of 0.6–0.8 were used, as seen in Table 1. The rationale for using values less than 1 in both ELS and SS is to provide the optimizer with more spots with the goal of improving dose conformality.

In this work, we copied each clinical plan and created four additional plans, with ELS scale factor set to 1.0, 1.2, and 1.4, and SS scale factor set to 1.0. The four plans were optimized with all other parameters kept the same as the clinical plans, including all the dosimetric objectives, robustness parameters, ion/spot for the initial spot dose calculation, and the MC statistical uncertainty in final dose calculation. The only difference in the four additional plans were the ELS or SS settings.

### 2.4 Plan delivery time

During the patient treatment sessions, the radiation was paused when the patients needed to take a break and re‐establish the breath‐hold, therefore the treatment delivery time can be much longer than the actual beam‐on time. For a direct comparison of the beam delivery time, all plans, including the clinical plans, were delivered on the ProBeam proton machine in QA mode, and the beam‐on time were recorded with a stopwatch. The QA mode doesn't require the respiratory gating signal; therefore, we can measure the actual beam delivery time.

### 2.5 Dosimetric comparison

To evaluate the dosimetric quality of the plans created with different ELS and SS, we compared the doses to the target, CTV D_98%_ on nominal plan and worst‐case scenario D_95%_ of the robustness. For the OARs, we extracted the D_0.03cc_, D_1cc_, and D_5cc_ for the large bowel, the small bowel, and the duodenum; D_0.03cc_ and D_mean_ for the esophagus and the heart; D_0.03cc_, D_5cc_, and V_45GyRBE_ for the stomach; D_mean_ to the liver (subtracting GTV); D_max_ to the spinal cord; D_mean_, V_20GyRBE_, V_5GyRBE_ to the lungs, and D_mean_ to the irradiated volume, which is the volume created by the 10% isodose line expanded by a 2 cm margin and limited to the patient body. Paired 2‐tail *t*‐test was used to compare the clinical plan to each investigated plan, with a *p*‐value less than 0.05 considered statistically significant.

## 3 RESULTS

### 3.1 Spot placement parameters

**FIGURE 1:**
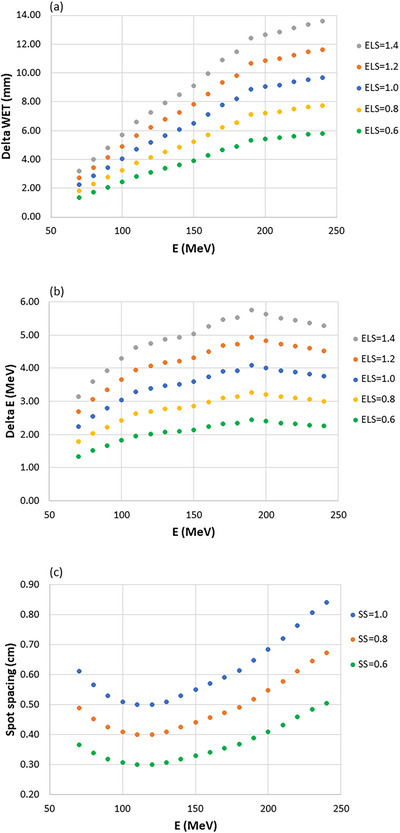
(a) The energy layer spacing (ELS) values between two adjacent layers in water‐equivalent‐thickness (WET) as a function of the proton energy when the ELS scale factors set to 0.6, 0.8, 1.0, 1.2, and 1.4 in the treatment planning system (TPS). (b) The same function as (a) plotted in energy difference (MeV). (c) The spot spacing as a function of the proton energy with the scale factors set to 0.6, 0.8, and 1.0.

The default ELS (scale factor equals 1.0) is energy‐dependent and varies from to 9.70 mm water equivalent thickness (WET), or 2.24–4.10 MeV of the proton energy. When a scale factor is used, the ELS varies linearly to the factor, examples of the ELS values were plotted in Figure 1a,b with scale factor equals 0.6, 0.8, 1.0, 1.2, and 1.4. Similarly, the SS changes from 5.0 to 8.4 mm with scale factor 1.0; the values are shown in Figure 1c, together with scale factors 0.6 and 0.8.

### 3.2 Dosimetric quality

The dose statistics of the seven clinical plans were listed in Table 2. All plans achieved at least the prescribed dose to 98% of the CTV volumes on the nominal dose distribution. On the robust evaluation, the worst‐case scenario was greater than 95% volume receiving 95% of the prescribed dose. Note that not all OAR doses were available in all plans, as some OARs only receiving near‐zero dose, and were excluded from the dosimetric analysis.

**TABLE 2:** Dose statistics of the clinical plans for the clinical target volume (CTV) and the organ‐at‐risks (OARs). The irradiated volume is the tissue within patient's body and within 2 cm distance from the 10% isodose line. The robustness is evaluated with the worst‐case scenario (w.c.s) considering combined 5‐mm setup up error and 3.5% range uncertainty (5.0% for lung: patients 5 and 7)

		Patient number						
ROI	Dose metrics	1	2	3	4	5	6	7
CTV	D_98%_ (%)	100.0	100.7	101.5	102.4	100.4	100.5	101.8
	D_98%_ (GyRBE)	50.4	52.9	50.7	59.4	60.3	58.4	71.3
	w.c.s. D_95%_(%)	99.0	95.3	96.5	100.9	98.4	98.9	100.9
	w.c.s. D_95%_ (GyRBE)	49.9	50.0	48.2	58.6	59.0	57.4	70.6
Irrad. vol.	D_mean_ (GyRBE)	9.9	13.0	8.1	12.1	13.2	9.6	12.9
Bowel large	D_0.03cc_ (GyRBE)	27.3	47.5	44.8	–	–	29.2	–
	D_1cc_ (GyRBE)	22.3	42.1	40.9	–	–	21.8	–
	D_5cc_ (GyRBE)	14.0	31.9	34.1	–	–	17.1	–
Bowel small	D_0.03cc_ (GyRBE)	50.0	3.0	35.1	1.2	–	–	–
	D_1cc_ (GyRBE)	44.5	0.6	30.1	0.3	–	–	–
	D_5cc_ (GyRBE)	30.1	0.1	19.9	0.1	–	–	–
Duodenum	D_0.03cc_ (GyRBE)	4.3	48.3	22.4	47.2	–	42.3	–
	D_1cc_ (GyRBE)	0.5	39.7	14.4	25.0	–	20.5	–
	D_5cc_ (GyRBE)	0.2	18.7	1.4	5.9	–	3.4	–
Esophagus	D_mean_ (GyRBE)	–	–	9.5	–	31.0	–	21.1
	D_0.03cc_ (GyRBE)	–	–	52.1	–	62.0	–	73.2
Heart	D_mean_ (GyRBE)	10.3	–	6.0	–	4.2	–	6.8
	D_0.03cc_ (GyRBE)	53.4	–	52.6	–	62.1	–	73.3
Liver‐GTV	D_mean_ (GyRBE)	1.0	13.4	2.1	10.6	–	14.4	5.8
Lungs	D_mean_ (GyRBE)	2.7	–		3.0	5.8	–	14.7
	V_20Gy_ (%)	4.0	–		6.6	11.2	–	30.9
	V_5Gy_ (%)	12.7	–		10.3	20.7	–	42.2
Spinal cord	D_max_ (GyRBE)	28.2	–	21.0	17.1	39.3	–	25.3
Spinal cord PRV	D_max_ (GyRBE)	33.7	–	25.2	19.6	42.9	–	29.9
Stomach	D_0.03cc_ (GyRBE)	51.6	47.1	52.9	–	–	6.2	–
	D_5cc_ (GyRBE)	50.4	38.7	52.2	–	–	1.2	–
	V_45GyRBE_ (cc)	26.9	0.5	50.6	–	–	0.1	–

Table 3 shows the difference in the doses for the four plans with increased ELS or SS to the clinical plans. Compared to the clinical plans, increasing ELS or SS did not cause clinically meaningful target coverage reduction (nominal or worst‐case scenario). Increasing ELS to 1.0, 1.2, and 1.4 didn't make a statistically significant difference on the OAR doses or the integral dose (mean dose to the irradiated volume), while increasing SS only slightly increased the integral dose and the mean dose to the normal liver.

**TABLE 3:** Dose differences to the clinical plans with the energy layer spacing (ELS) equals to 1.0, 1.2, 1.4, and spot spacing (SS) set to 1.0. Asterisks (*) indicates the difference is statistically significant (*p*‐value < 0.05)

		ELS1.0		ELS1.2		ELS1.4		SS1.0	
ROI	Dose metrics	AVG	Std.	AVG	Std.	AVG	Std.	AVG	Std.
CTV	D_98%_ (%)	−0.2	0.4	−0.1	0.4	−0.3	0.5	−0.2	0.5
	D_98%_ (Gy)	−0.1	0.2	−0.1	0.2	−0.2	0.3	−0.1	0.3
	w.c.s. D_95%_(%)	0.0	0.2	0.1	0.2	−0.1	0.5	−0.1	0.4
	w.c.s. D_95%_ (GyRBE)	0.0	0.1	0.0	0.1	−0.1	0.3	−0.0	0.2
Irrad. vol.	Dmean (GyRBE)	−0.1	0.1	−0.0	0.1	0.0	0.1	0.1*	0.1*
Bowel large	D_0.03cc_ (GyRBE)	0.1	0.2	0.2	0.2	0.1	0.6	0.5	0.7
	D_1cc_ (GyRBE)	−0.2	0.3	−0.1	0.1	−0.2	0.3	0.0	0.5
	D_5cc_ (GyRBE)	−0.3	0.2	−0.1	0.4	−0.2	0.3	0.0	0.2
Bowel small	D_0.03cc_ (GyRBE)	−0.4	0.4	−0.3	0.4	−0.3	0.8	−0.2	0.3
	D_1cc_ (GyRBE)	−0.4	0.5	0.0	0.2	−0.6	0.9	0.1	0.7
	D_5cc_ (GyRBE)	−0.5	0.7	0.1	0.3	−1.1	2.3	0.0	0.6
Duodenum	D_0.03cc_ (GyRBE)	−0.4	1.1	−0.6	2.1	−0.0	1.5	−0.2	0.7
	D_1cc_ (GyRBE)	0.0	0.9	0.4	0.9	0.9	1.6	0.5	1.3
	D_5cc_ (GyRBE)	−0.7	1.9	−0.3	1.4	−0.3	2.1	0.3	0.6
Esophagus	D_mean_ (GyRBE)	−0.0	0.0	0.1	0.2	0.1	0.1	0.1	0.2
	D_0.03cc_ (GyRBE)	−0.0	0.1	−0.0	0.2	−0.1	0.1	0.2	0.2
Heart	D_mean_ (GyRBE)	−0.1	0.2	−0.1	0.2	0.0	0.1	0.1	0.1
	D_0.03cc_ (GyRBE)	−0.1	0.2	−0.1	0.2	−0.1	0.3	−0.0	0.3
Liver‐GTV	D_mean_ (GyRBE)	−0.1	0.2	−0.0	0.2	0.0	0.2	0.2*	0.1*
Lungs	D_mean_ (GyRBE)	0.0	0.1	0.1	0.1	0.1	0.1	0.1	0.1
	V_20Gy_ (%)	0.0	0.1	0.1	0.2	0.1	0.2	0.2	0.4
	V_5Gy_ (%)	0.1	0.2	0.2	0.3	0.2	0.2	0.4	0.2
Spinal cord	D_max_ (GyRBE)	−0.1	0.3	−0.1	0.2	0.1	0.2	−0.1	0.3
Spinal cord PRV	D_max_ (GyRBE)	−0.2	0.4	−0.3	0.4	0.2	0.5	−0.4	0.5
Stomach	D_0.03cc_ (GyRBE)	−0.2	0.3	−0.3	0.5	0.1	0.4	0.1	0.5
	D_5cc_ (GyRBE)	−0.0	0.2	0.1	0.2	−0.0	0.1	0.0	0.2
	V_45GyRBE_ (cc)	0.2	0.6	0.0	0.2	0.0	0.3	0.5	1.4

**FIGURE 2:**
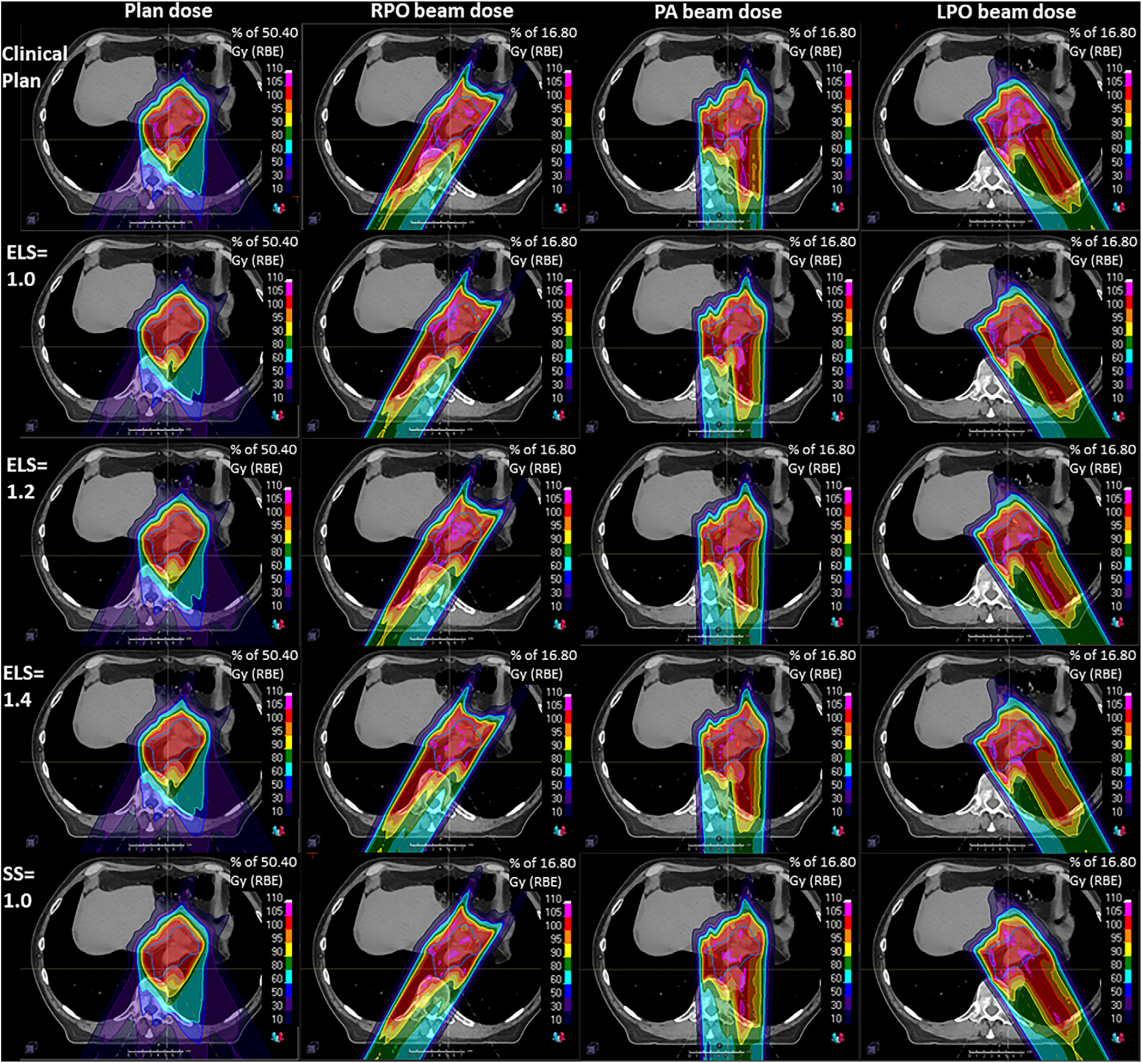
Dose distribution of patient 1. From left to right: plan dose, right‐posterior oblique beam dose, posterior‐anterior beam dose, and left‐posterior oblique beam dose. From top to bottom: clinic plan, and reoptimized plan with ELS = 1.0, ELS = 1.2, ELS = 1.4, and SS = 1.0, respectively. ELS, energy layer spacing; SS, spot spacing.

An example of the plan and beam dose distributions are displayed in Figure 2. The target was covered by three beams, each was optimized to deliver a uniform dose using single field optimization (SFO). Although the beam doses showed slightly different homogeneity, the plan dose distribution didn't reveal any clinically relevant difference among the five plans. Such similarity of plan and beam dose distribution was observed for all the patients in this study.

### 3.3 Delivery time

The measured beam delivery time of the 35 plans (130 fields total) are shown in Table 4. Compared to the clinic plans, with the ELS values increased to 1.0, 1.2, and 1.4, the beam delivery time decreased by 9.2 ± 3.4 s (18.7 ± 5.8%), 11.6 ± 3.5 s (23.1 ± 5.9%), and 14.7 ± 4.0 s (28.9 ± 6.1%), respectively. The delivery time was reduced by 1.1 ± 1.4 s (1.9 ± 2.9%) with the SS increased to 1.0.

**TABLE 4:** Measured delivery time for the 130 fields. The average and standard deviation of the time for each plan type is listed at the bottom. The time reductions from the clinical plan are calculated for the four plans with increased energy layer spacing (ELS) or spot spacing (SS). Asterisks (*) indicate the difference is statistically significant (*p*‐value < 0.05)

		Measured time (second)				
Patient #	Field #	Clinic plan	ELS 1.0	ELS 1.2	ELS 1.4	SS 1.0
1	1	66.7	51.4	47.9	44.0	64.4
	2	66.0	49.2	48.2	43.3	62.2
	3	59.3	47.3	44.4	40.2	59.0
2	1	61.0	50.0	46.7	44.7	59.1
	2	61.6	50.2	48.1	44.4	56.8
	3	56.7	46.5	46.2	42.5	54.1
3	1	51.3	37.2	35.3	31.8	50.1
	2	36.4	29.1	25.3	22.2	35.7
	3	39.1	30.5	28.8	24.1	40.2
	4	52.3	38.6	35.6	32.4	52.1
	5	45.3	33.3	31.1	27.1	43.8
4	1	44.7	36.2	36.9	35.1	43.2
	2	45.6	37.4	35.8	33.8	44.5
	3	43.6	38.3	37.9	35.1	43.9
5	1	52.2	45.4	41.5	38.6	49.8
	2	46.8	41.2	38.1	36.0	48.1
	3	45.1	41.6	37.7	34.7	45.4
	4	48.8	40.2	39.0	36.3	49.0
	5	49.5	44.8	42.1	37.7	50.0
6	1	34.6	27.5	26.6	24.7	34.1
	2	37.9	28.7	25.0	23.9	36.1
	3	34.1	28.5	26.1	24.2	33.7
7	1	41.2	33.6	30.7	26.9	40.8
	2	48.5	38.9	35.0	33.0	46.9
	3	50.4	41.7	37.3	33.6	48.1
	4	40.1	33.0	30.7	27.5	40.0
Average (s)		48.4	39.2	36.9	33.8	47.3
Std.		9.2	7.4	7.4	7.0	8.5
Time reduction (s)			9.2 ± 3.4*	11.6 ± 3.5*	14.7 ± 4.0*	1.1 ± 1.4
Time reduction (%)			18.7 ± 5.8	23.1 ± 5.9	28.9 ± 6.1	1.9 ± 2.9

**FIGURE 3:**
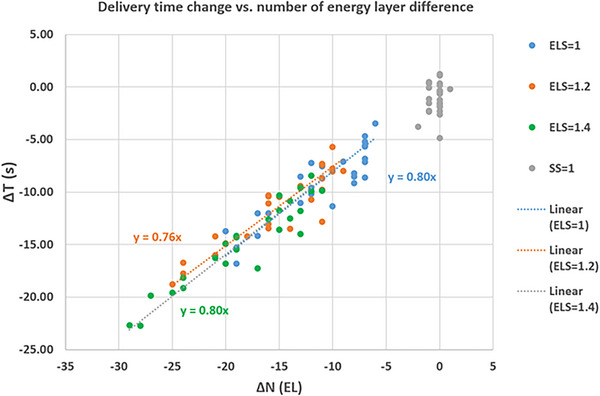
The relationship between the change of delivery time versus the number of energy layers when the energy layer spacing (ELS) or spot spacing (SS) are set to larger values compared to the clinic plans. The linear fitting is also plotted for plans with ELS set to 1.0, 1.2, and 1.4.

With the increased ELS, the number of energy layers in the beams decreased. Figure 3 plotted the reduction of beam delivery time as function of number of energy layers reduced (from the clinical plan) for the ELS values of 1.0, 1.2, and 1.4. Linear regression of the three data series indicated a time reduction of 0.80, 0.76, and 0.80 s/layer, respectively. Increasing SS, in principle, doesn't affect number of energy layers in the initial spot placement. In reality, a small number of energy layers may be removed when the low‐weight spots were removed from the plan to meet the machine delivery limit. As also shown in Figure 3, setting SS to 1.0 resulted in 1−2 energy layers difference, and no trend of delivery time reduction was observed.

## 4 DISCUSSION

The IMPT plan creation is an inverse optimization process. In commercial treatment planning systems, it starts with predetermined initial proton spot distributions, that is, the energy and the location of the proton spots are fixed, only the spot weighting (MU) is changing during the optimization to achieve the desired dose distribution. For a particular target and beam direction, a smaller ELS and/or SS results in more spots in the beam, providing more variables in the optimization; therefore, in theory, should allow closer to ideal dose distribution.

However, using too many layers or spots can be problematic. First, there are inherent limits of what a clinical proton machine can deliver with acceptable accuracy. Typical cyclotron‐based clinical proton systems can't deliver spots with MU less than the minimum limit, therefore, for a patient plan to be deliverable, low‐MU spots need to be removed or increased to at least the minimum MU. This will alter the optimized spot weights and degrade the final dose distribution. Second, it takes time to switch from one layer to the next. More layers require more time for layer switch and increase the total beam‐on time. For spot scanning systems, the beam is turned off after every spot; more spots mean longer total dead time and longer beam delivery time. Finally, when treating mobile targets with breath‐hold, longer beam delivery time could result in more breath‐hold cycles, further increasing overall patient on table time and potentially cause breath‐hold fatigue.

In fact, there is a range of ELS and SS values that allow acceptable clinical plans, as inverse optimization is a highly degenerated problem,^16^, ^17^, ^18^ especially for IMPT plan optimization.^1^, ^19^, ^20^, ^21^ For example, Muller et al., utilized a multiple‐optimization method to reduce the number of energy layers and showed reduction of delivery time of 28% without major degradation of plan quality.^13^ Using an iterative pencil beam resampling technique to optimize the number of energy layers and spots, van de Water et al. achieved 95% of spot reduction and shortened the delivery time by 46% for a head and neck IMPT plan without compromising plan quality.^11^

Previous works required dedicated research tools developed by individual research group, which are not available to most users. In the contrast, we directly changed the setting for the initial ELS in a commercial TPS. As demonstrated in this work, there is no clinically relevant dose difference among the treatment plans when the ELS factor is increased to 1.0, 1.2, and 1.4, while the beam delivery time is decreased by 18.7% (9.2 s), 23.1% (11.6 s), and 28.9% (14.7 s), respectively. The beam delivery time reduction is roughly proportional to the number of energy layer change, and the rate of ∼0.8 s/layer is consistent with the energy layer switch time of our proton system.

The increased SS didn't introduce plan quality degradation, except a slightly higher integral dose. With larger SS, the spots are placed further outside of the target, which results in broader penumbra and therefore caused slightly higher integral dose. Increasing the SS didn't affect the beam delivery time, as it is apparent that the number of energy layers barely changed. The spot switching time is different for the spot scanning, raster scanning, and the line scanning technique.^22^ The raster scanning system is used for our ProBeam system; within each energy layer, the beam remains on when moving from one spot to the next if the distance between the two spots is below the threshold (10 mm). When the beam is turned off between spots, the dead time is 3 ms. In our plans, the SS is less than 10 mm, as seen in Figure 1; therefore, the number of spots hardly has an effect of the total delivery time. For spot scanning systems, the dead time (3 ms) between adjacent spots can have more effect on the delivery time, especially for plans with a substantially large number of spots.

Our study was conducted for a specific cyclotron‐based proton delivery system; however, we believe the results are applicable to other cyclotron or synchrotron systems, as the amount of time reduction is proportional to the layer switch time. As Cao et al.,^12^ pointed out, the layer switch time for their synchrotron proton system is 2.1 s, much longer than the 0.8 s for our cyclotron‐based system. The potential delivery time shortening from the reduction of energy layers can be even more meaningful. The optimal ELS, though, is dependent on the Bragg peak width and can be different than the results of this study.

It is very easy to change the initial ELS and SS in our treatment planning system, RayStation, as it allows the user to change these values for each beam. Based on the results of this study, we now use ELS value of 1.4 for all the IMPT plans with breath‐hold. The other commonly used TPS for IMPT planning is Eclipse, it allows changing ELS and SS parameters for each beam model, rather than individual plans. An alternative for Eclipse user is to create multiple beam models with different ELS and/or SS settings and choose the corresponding beam model as desired.

## 5 CONCLUSION

We demonstrated that reducing the number of energy layers can effectively reduce the beam delivery time, while reducing the number of spots per layer had no effect. No difference in plan quality was observed with ELS variation, while larger SS results in a slightly higher dose to normal tissue. We suggest that proton machine users search for the optimal energy layer and SS for their specific machine characteristics to achieve the balance between the delivery efficiency and plan quality for IMPT treatments.

## AUTHOR CONTRIBUTIONS

Mingyao Zhu: Conceptualization, design of the work, data analysis and interpretation, and writing the original manuscript. Stella Flampouri: Data analysis and interpretation, reviewing, and editing manuscript. Alex Stanforth: Data acquisition, reviewing, and editing manuscript. Roelf Slopsema: Data analysis and interpretation, reviewing, and editing manuscript. Zachary Diamond: Data acquisition, reviewing, and editing manuscript. William LePain: Data acquisition, reviewing, and editing manuscript. Katja Langen: Conceptualization, design of the work, data interpretation, reviewing, and editing manuscript.

## CONFLICT OF INTEREST STATEMENT

Katja Langen reports in‐kind loan of research software from RaySearch. No other conflicts of interest related to the submitted work.
